# Magnesium rescues the morphology of *Bacillus subtilis mreB* mutants through its inhibitory effect on peptidoglycan hydrolases

**DOI:** 10.1038/s41598-021-04294-5

**Published:** 2022-01-21

**Authors:** Benoit Tesson, Alex Dajkovic, Ruth Keary, Christian Marlière, Christine C. Dupont-Gillain, Rut Carballido-López

**Affiliations:** 1 Micalis Institute, INRAE, AgroParisTech, Université Paris-Saclay, 78350 Jouy-en-Josas, France; 2 Laboratoire de Physique des Solides, LPS, University Paris-Saclay, CNRS, Orsay, France; 3 Bio- and Soft Matter Division, Institute of Condensed Matter and Nanosciences, Université catholique de Louvain, 1348 Louvain-la-Neuve, Belgium; 4 Present Address: Biomillenia (Design Pharmaceuticals), BIOCITECH, 93230 Romainville, France

**Keywords:** Bacteria, Cellular microbiology, Cell growth

## Abstract

Cell wall homeostasis in bacteria is tightly regulated by balanced synthesis and degradation of peptidoglycan (PG), allowing cells to expand their sacculus during growth while maintaining physical integrity. In rod-shaped bacteria, actin-like MreB proteins are key players of the PG elongation machinery known as the Rod complex. In the Gram-positive model bacterium *Bacillus subtilis* depletion of the essential MreB leads to loss of rod shape and cell lysis. However, millimolar concentrations of magnesium in the growth medium rescue the viability and morphological defects of *mreB* mutants by an unknown mechanism. Here, we used a combination of cytological, biochemical and biophysical approaches to investigate the cell surface properties of *mreB* null mutant cells and the interactions of Mg^2+^ with the cell wall of *B. subtilis*. We show that ∆*mreB* cells have rougher and softer surfaces, and changes in PG composition indicative of increased DL- and DD-endopeptidase activities as well as increased deacetylation of the sugar moieties. Increase in DL-endopeptidase activity is mitigated by excess Mg^2+^ while DD-endopeptidase activity remains high. Visualization of PG degradation in pulse-chase experiments showed anisotropic PG hydrolase activity along the sidewalls of *∆mreB* cells, in particular at the sites of increased cell width and bulging, while PG synthesis remained isotropic. Overall, our data support a model in which divalent cations maintain rod shape in ∆*mreB* cells by inhibiting PG hydrolases, possibly through the formation of crosslinks with carboxyl groups of the PG meshwork that affect the capacity of PG hydrolases to act on their substrate.

## Introduction

Most bacteria are surrounded by a macromolecular structure named the cell wall (CW), which gives rigidity and mechanical protection and is a major determinant of cell shape. The CW is primarily composed of the polymer peptidoglycan (PG), a meshwork of rigid linear strands of alternating subunits of N-acetyl glucosamine and N-acetyl muramic acid cross-linked by flexible peptide stems. The stem peptides are attached to N-acetyl muramic acid and are synthesized as pentapeptide chains. The pentapeptide sequence varies across species and has the particularity of involving d-amino acids. In the rod-shaped Gram-positive model bacterium *Bacillus subtilis,* the pentapeptide contains l-alanine (l-Ala) in the first position, followed by d-isoglutamic acid (d-iGlu), *meso*-diaminopimelic acid (mDAP) and two terminal d-alanines (d-Ala). In most bacteria including *B. subtilis* the main cross-linking strategy yields to a 4 → 3 crosslink (DD-transpeptidation, catalyzed by penicillin binding proteins, PBPs), which connects the carbonyl moiety of the 4th amino-acid (d-Ala) of the acyl-donor stem peptide to the amino group of the 3rd amino-acid (mDAP) of the acyl-acceptor stem peptide and uses disaccharide-pentapeptides as substrate. Cell walls of Gram-positive bacteria further comprise phosphate-containing anionic polymers, the teichoic acids (TAs), which can be either covalently linked to PG (wall teichoic acids, WTAs) or anchored to the cytoplasmic membrane (lipoteichoic acids, LTAs) and have important regulatory functions^1,2^.

Growth of the PG sacculus depends on the coordinated action of PG synthases, which make and crosslink new glycan chains (transglycosylases and transpeptidases, respectively), and PG hydrolases, which cut existing bonds to allow sacculus maturation and expansion^3^. PG hydrolases possess a wide range of hydrolytic activities and cleave bonds either within the sugar moieties of the glycan chains (glucosaminidases and lytic transglycosylases), between the N-acetyl muramic acid and the stem peptide (amidases) or between amino acids in the peptide stems or the peptide bridges (peptidases)^4,5^. Peptidases can cleave between two d-amino acids (dd-peptidases) or between an l- and a d-amino acid (ld- and dl-peptidases), and are further classified as carboxypeptidases or endopeptidases depending on whether they remove the C-terminal amino acid of the stem peptide (caboxypeptidases) or they cut between two amino acids inside the peptide stems or bridges (endopeptidases)^4,5^. In total, > 40 definite or putative PG hydrolases have been identified in *B. subtilis.* Such multiplicity and functional redundancy together with complex regulation mechanisms hampers the study of their individual roles^4–6^*.* Uncontrolled activity of PG hydrolytic enzymes can compromise cellular integrity. For example, when the proton motive force is dissipated by depolarizing agents, certain PG hydrolases known as autolysins are activated and lyse the cells^7^. The potentially lethal activity of these enzymes must therefore be tightly regulated. The functions of known PG hydrolases are generally consistent with their transcriptional profiles, but the mechanisms involved in post-translational control of their activities remain largely unknown^4^. Proposed mechanisms include the CW ionic and pH environment, the proton motive force, protein–protein interactions, extracellular proteases activity, controlled transport across the cytoplasmic membrane, interaction with TAs and local modification (conformational change or covalent modification) of the PG substrate. Covalent modifications of PG known to affect PG hydrolases activity include deacetylation of N-acetyl glucosamine residues^8^, O-acetylation of N-acetyl muramic acid^9^ and amidation of mDAP^10^. Similarly, addition of protonated d-alanine ester linkages (D-alanylation) to TAs has been shown to affect PG hydrolase activity^11–14^.

Proteins of the MreB family are structural homologues of eukaryotic actin that are highly conserved and essential in non-spherical bacteria. They are thought to form scaffolding polymers that organize and orient the movement of PG synthesizing enzymes in the membrane in order to allow controlled cylindrical expansion^15,16^. In *B. subtilis,* MreB isoforms have also been shown to regulate the activity of LytE and CwlO, two co-essential dl-endopeptidases required for sidewall elongation^17–20^.

Magnesium plays a key role in *B. subtilis* CW homeostasis. Millimolar concentrations of Mg^2+^ in the growth medium are known to rescue the viability and/or rod-shape morphology of several CW-related mutants including *mreB* mutants^21,22^. Several hypotheses have been put forward regarding the rescue role of Mg^2+^: it could affect the activity or the stability of CW enzymes; strengthen the sacculus by crosslinking negatively charged groups, and/or stabilize the cytoplasmic membrane and thus associated PG biosynthetic complexes^21^. Importantly, we recently showed that in wild-type *B. subtilis* cells, excess Mg^2+^ leads to a decrease in the amidation of mDAP in the PG and inhibits at least some PG hydrolases involved in autolysis^10^. Here, we show that the morphological defects of Δ*mreB* cells are due to dysregulated PG hydrolase activity, and that excess Mg^2+^ restores viability and rod shape by inhibiting PG hydrolases.

## Results

### *mreB* null mutant cells have rougher and softer cell surfaces

**Figure 1:**
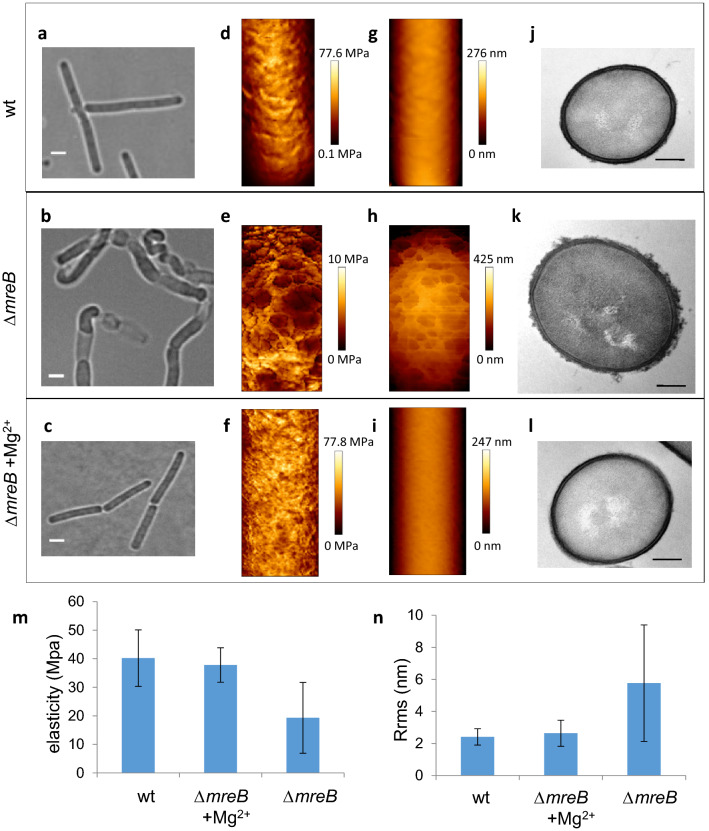
*mreB* mutant cells have rougher and softer cell walls. Cell wall structure and mechanical properties of wild-type and Δ*mreB* cells exponentially growing in LB medium in the presence and in the absence of 25 mM MgSO_4_. (**a**–**c**) phase contrast images. Scale bars, 2 µm. (**d**–**i**) AFM elasticity map (**d**–**f**) and topography (**g**–**i**). AFM scan size: wild-type, 0.75*1.71 µm^2^; Δ*mreB,* 0.81*1.67 µm^2^; Δ*mreB* + Mg^2+^, 0.69*1.51 µm^2^. (**j-l**) Representative TEM transversal sections. Scale bars, 0.2 µm. (**m**, **n**) Average elasticity (**m**) and roughness (**n**) of the cell envelope determined from the AFM analyses. Bars indicate standard deviation.

In the absence of *mreB*, *B. subtilis* cells lose control over their width, bulge and eventually lyse (Fig. 1a,b, and Movie 1)^22^. However, when 5–25 mM Mg^2+^ is added to the growth medium, cells maintain a rod shape and no lysis occurs (Fig. 1c)^22^. To gain insight into the mechanisms underlying this phenotype, we first used atomic force microscopy (AFM) to investigate the cell surface structure and mechanical properties of ∆*mreB* mutant cells with minimal perturbation. Cells growing exponentially in the presence and in the absence of 25 mM MgSO_4_ were immobilized and analyzed in their growth medium. Simultaneous cartography of the topography and of the mechanical properties of the cell surface was obtained using the Quantitative Imaging technique. Using this approach, we recently showed that excess Mg^2+^ has no effect on the rigidity or the nanoscale roughness of wild-type *B. subtilis* cells^10^. In the presence of excess Mg^2+^, the mechanical properties of ∆*mreB* cells did not exhibit any apparent difference relative to wild-type cells, with a Young modulus of 37.8 MPa (sd, 6.0) and 40.2 MPa (sd, 9.9) respectively (Fig. 1d,f,m). The topography of the cell surface, quantified from height profiles (Supplementary Fig. S1) and root mean square roughness (Rrms), was also very similar in wild-type cells and ∆*mreB* mutant cells grown in the presence of high Mg^2+^ (Rrms = 2.6 ± 0.8 nm and 2.4 ± 0.6 nm, respectively) (Fig. 1g,i,n). In contrast, ∆*mreB* cells grown without supplemented Mg^2+^ displayed increased roughness and lower rigidity. Young’s moduli varied greatly between cells depending on the severity of their morphological deformations, with an average of 19.3 MPa (sd, 12.4) (Fig. 1e,m). Surface roughness increased to 5.8 nm ± 3.6, about twofold relative to cells grown in the presence of excess Mg^2+^ (Fig. 1h,n, Supplementary Fig. S1).

To complement the AFM measurements, we imaged the cell envelope at high resolution by transmission electron microscopy (TEM). In the presence of excess Mg^2+^, the sidewalls of *∆mreB* mutant cells presented a homogeneous, smooth structure, like wild-type cells (Fig. 1l and j, respectively, and Supplementary Fig. S2). In contrast, *∆mreB* cells grown without added Mg^2+^ displayed an irregular, rough cell surface. A feature of these cells was lower density of electron staining of their walls, which might suggest decreased packing density. Another striking difference was that the outer CW surface contained large amounts of ragged material, with fragments ‘peeling off’ and giving a fibrous appearance (Fig. 1k and Supplementary Fig. S2). Interestingly, multiple lytic enzyme mutants of *B. subtilis*^23^ and *ugtP* mutant cells, which have increased dl-endopeptidase activity^24^, display similar rougher walls, interpreted as partially degraded PG material.

Taken together, our AFM and TEM results suggest that *mreB* mutant cells have a weakened CW undergoing higher or uncontrolled degradation.

### The elemental composition of the cell surface is altered in Δ*mreB* cells and in the presence of magnesium

**Figure 2:**
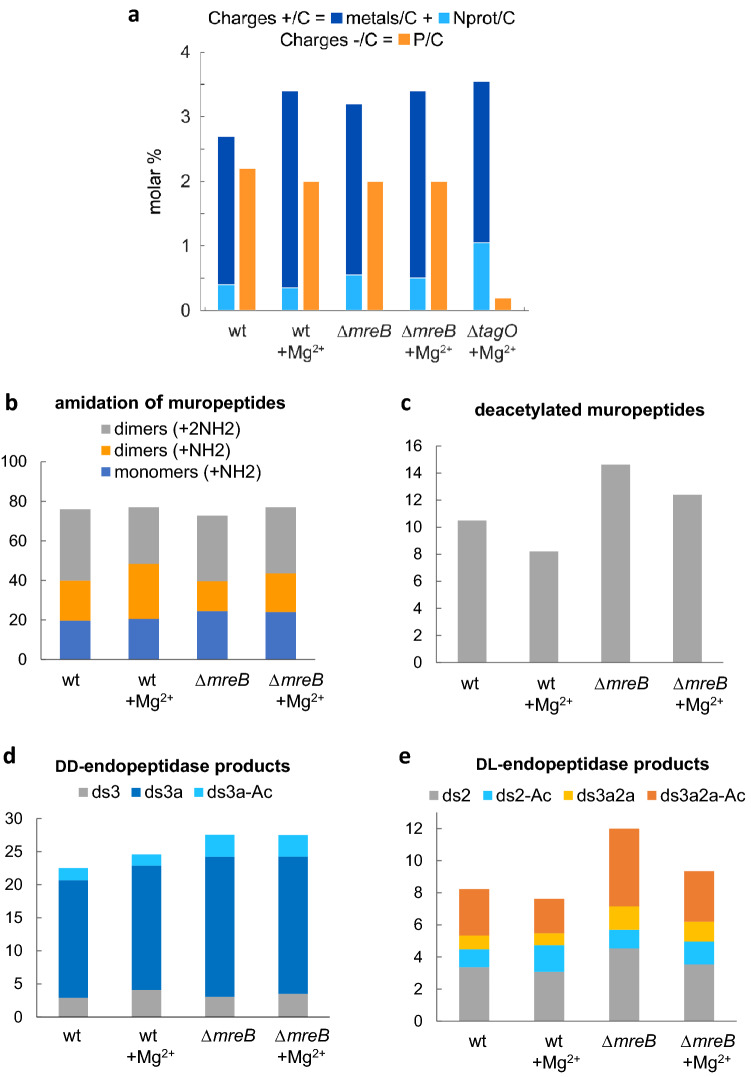
Chemical changes in the cell walls of *mreB* mutant cells. (**a**) Relative quantification of charges at the cell surface of wild–type, ∆*mreB* and ∆*tagO* cells. Charges detected by XPS expressed as molar percentage. Cells were grown in LB medium with or without added Mg^2+^ as indicated. % Total positive charges = % total metals (Na + K + 2·Ca + 2·Mg) + % protonated amines (Nprot). % Total negative charges = % phosphorus . The average of two independent sets of experiments (Table S2) is presented for each condition (see Methods for details of the quantifications). (**b**–**e**) Percentage of amidated muropeptides (**b**), deacetylation of muropeptides (**c**), DD-endopeptidase products (**d**) and DL-endopeptidase products (**e**) in wild-type and ∆*mreB* mutants grown with and without excess magnesium. Percentages were calculated from the quantification of the UPLC peaks (see Materials and Methods). Muropeptide composition analysis were performed in duplicate for all strains. ds refers to disaccharide (GlcNAc-MurNAc) and is followed by numbers which indicate the peptide length, as follows: 2 = dipeptide (L-Ala-D-iGlu), 3 = tripeptide (L-Ala-D-iGlu-mDAP). (a) = amidation. (-Ac) = missing acetyl group.

To test whether the CW defects of Δ*mreB* cells are accompanied by changes in the elemental composition of the cell surface, we analyzed the surface composition of intact freeze-dried cells using X-ray Photoelectron Spectroscopy (XPS). This technique measures the elemental and functional composition of the outermost surface of a sample within a few nanometers (≤ 10 nm)^25^. In cells of the well characterized *B. subtilis* laboratory strain 168, which lack the capsule and the S-layer, this outermost surface contains PG and TAs. The main elements detected were, in order of importance: carbon, oxygen, nitrogen, phosphorus and physiologically relevant group I and II metals (sodium, magnesium, calcium and potassium) (Table 1). Phosphorus in the CW is mainly attributed to TAs. In agreement with this, when production of WTAs was abolished in a Δ*tagO* null mutant^26^, the level of phosphorus decreased dramatically (Fig. 2a and Table 1). MreB proteins have been suggested to have a role in WTA synthesis or attachment^27,28^. However, phosphorus was present in similar amounts in the wild-type and the ∆*mreB* mutant, regardless of the presence of excess Mg^2+^ (Fig. 2a and Table 1), indicating that the amount of TAs in the cell surface is not affected in the absence of *mreB*. Nitrogen is mainly attributed to PG (sugar moiety or stem peptides). The levels of nitrogen were significantly increased in the Δ*tagO* mutant (Table 1). Since XPS is a relative quantification technique, this is nevertheless due to the absence of phosphorus (WTA), leading to a proportional increase of PG at the cell surface. However, the CW of the ∆*mreB* mutant was enriched in nitrogen while phosphorous levels were comparable to wild-type (Table 1), suggesting modifications in the chemistry of PG in the absence of *mreB*.

**Table 1:** Elemental cell surface composition determined by XPS for the wild-type, the ∆*mreB* and the ∆*tagO* mutants grown in LB medium supplemented or not with 25 mM Mg^2+^.

	C	O	N	P	Mg	K	Na	Ca
wt	65,7	25,3	4,9	2,4	0,3	0,1	1,1	0,1
	66,0	25,1	4,6	2,2	0,5	0,1	1,3	bdl
wt + Mg^2+^	72,7	21,2	2,7	1,7	0,9	bdl	0,2	0,6
	64,6	27,2	4,4	2,0	0,9	0,2	0,6	0,1
Δ*mreB*	62,1	26,7	6,0	2,4	0,3	0,9	1,5	0,1
	66,7	23,9	5,7	2,0	0,3	0,2	1,1	0,1
Δ*mreB* + Mg^2+^	59,0	31,0	5,2	2,9	1,3	bdl	0,6	0,1
	63,1	27,5	5,8	2,0	0,8	0,2	0,5	bdl
Δ*tagO* + Mg^2+^	66,3	23,5	8,6	0,2	0,9	bdl	0,4	bdl
	58,8	29,0	9,8	0,2	1,3	bdl	0,4	bdl

Note that the Δ*tagO* null mutant only grew in liquid LB in the presence of high Mg^2+^ (Supplementary Fig. S9a) and thus XPS analysis could only be performed at high Mg^2+^. Results are expressed as molar fractions (in %), excluding hydrogen. Results of two independent sets of experiments are presented. C, carbon; O, oxygen; N, nitrogen; P, phosphorus; Na, sodium; Mg, magnesium; Ca, calcium and K, potassium. bdl = below detection limit.

To further understand the interaction of Mg^2+^ with the CW and the balance of charges in the cell surface, we quantified the charges detected by XPS in the wild–type and the ∆*mreB* and ∆*tagO* mutants. The cell walls of Gram-positive bacteria are negatively charged and they bind metals^29,30^. Accordingly, Mg^2+^ as well as Na^+^, Ca^2+^ and K^+^ were present at the surface of *B. subtilis* cells grown with or without added Mg^2+^ (Table 1). In the presence of excess Mg^2+^, the total amount of bound metals increased as expected (Fig. 2a and Table S2). The amount of bound Mg^2+^ increased ~ threefold while the amount of bound Na^+^ decreased ~ twofold (Table 1), suggesting that the Mg^2+^ concentration in LB may not be sufficient to saturate all binding sites in the CW, contrary to previous estimates^31^. Charges present at the surface of *B. subtilis* at physiological pH are imputable to several chemical groups. Negative charges come from phosphate groups of TAs and from carboxyl groups of PG peptides. Positive charges come from protonated amines of TAs and PG (D-alanylated TAs and amidated PG stem peptides, respectively)^45^. The calculated amount of positive charges (metals + protonated amines) exceeded the amount of calculated negative charges coming from phosphate groups in all strains and conditions, in particular when cells were cultivated with excess Mg^2+^ (Fig. 2a and Table S2). This indicates that PG carboxyl groups also have an important role in metal binding in the *B. subtilis* CW, as previously reported^29,30^. The contribution to the XPS carbon peak at about 289 eV is attributed to both carboxylic acid and ester functions^46^ and thus the exact contribution of PG carboxyl groups could not be determined.

Taken together, our XPS data suggest that excess Mg^2+^ in the growth medium binds to PG carboxyl groups in addition to the classically appreciated TAs, and that the cell surface of ∆*mreB* mutant cells contains more nitrogen than the surface of wild-type cells. This increase in nitrogen amounts could be due to amidation of PG and/or to D-alanylation of TAs, or be a relative effect due to deacetylation of sugar residues. These three chemical modifications of the CW are thought to modulate the activity of PG hydrolases^8,10–14^.

### Endopeptidase activity is deregulated in Δ*mreB* cells

To examine possible chemical changes in the PG of the ∆*mreB* mutant, we analyzed the muropeptide composition of the PG of wild-type and ∆*mreB* cells grown in the presence or in the absence of excess Mg^2+^. Muropeptide profiles (Supplementary Fig. S3 and Table S1) were similar to those previously reported for growing wild-type *B. subtilis* cells^10,32,33^. As previously reported^10^, in wild-type cells excess extracellular Mg^2+^ caused a decrease in amidation of dimeric muropeptides, which in *B. subtilis* occurs in the mDAP stem peptide^32^. Dimeric muropeptides in which only one of the two mDAP residues is amidated increased, with a corresponding decrease of doubly amidated dimeric muropeptides (Fig. 2b, Supplementary Fig. S3 and Table S1)^10^. In ∆*mreB* cells, the number of doubly amidated dimers was only slightly affected but the number of singly amidated dimers significantly decreased (Fig. 2b and Supplementary Table S1). Noteworthy, in the presence of excess Mg^2+^ the decrease of single amidated dimers was compensated to reach the same level as in wild-type cells grown without Mg^2+^ (Fig. 2b and Supplementary Table S1).

The PG of *∆mreB* cells was also more deacetylated than wild-type PG (Fig. 2c and Supplementary Table S1). N-deacetylation is one of the most common modifications of PG in Gram-positive bacteria. It abolishes the binding of LysM-containing proteins such as certain PG hydrolases (e.g. LytE), and is therefore thought to modulate their activity^8^. Since we previously showed that Mg^2+^ inhibits PG hydrolases in wild-type *B. subtilis* cells^10^, it would be expected that deacetylation would be reduced when cells are grown in excess Mg^2+^ as cell homeostatic compensatory mechanism. This is precisely what we found (Fig. 2c and Supplementary Table S1).

The amount of disaccharide pentapeptide was increased in Δ*mreB* cells (Supplementary Table S1), indicating that the activity of dd-carboxypeptidases, which remove the terminal d-Ala residue from the pentapeptide chain of the PG precursor, is reduced. The main cross-linking strategy (4 → 3 crosslink) between glycan strands uses disaccharide pentapeptides as substrate. By removing the 5th amino-acid, dd-carboxypeptidases are thought to control the extent of cross-linking of the sacculus^34^. The cross-linking index was however not appreciably different in cells lacking *mreB* (Supplementary Table S1), suggesting that dd-carboxypeptidase activity may be reduced to compensate for the increased endopeptidase activity observed.

Importantly, PG of the ∆*mreB* mutant was also enriched in muropeptides resulting from endopeptidase activity, with almost 50% increase relative to the wild-type in the absence of Mg^2+^ and ~ 25% increase in the presence of excess Mg^2+^ (Supplementary Fig. S3 and Table S1). Activities of both DD-endopeptidases and DL-endopeptidases were affected by both Mg^2+^ and the absence of *mreB* (Fig. 2d and e, and Supplementary Table S1). DD-endopeptidase products increased slightly in wild-type cells grown in high Mg^2+^ and increased even more in ∆*mreB* cells (Fig. 2d). This increased activity of DD-endopeptidases was on the deacetylated and amidated forms of muropeptides in cells lacking *mreB*. Interestingly, the increase of DD-endopeptidase activity in ∆*mreB* cells was not compensated by excess Mg^2+^ (Fig. 2d). These results suggest that DD-endopeptidases are deregulated in the absence of *mreB,* but that the compensatory effect of magnesium on rescuing ∆*mreB* mutant cells is not mediated via effects on DD-endopeptidases.

In contrast to DD-endopeptidases, DL-endopeptidase activity was not significantly affected when wild-type cells were grown in the presence of excess Mg^2+^ (8.2% vs 7.6% of all muropeptides resulting from DL-endopeptidase activity, Fig. 2e), but it increased ~ 50% in *mreB* mutant cells grown without Mg^2+^ (to produce 12% of all muropeptides, Fig. 2e). All detected DL-endopeptidase products were affected except deacetylated disaccharide dipeptide (Fig. 2e). This contrasts with the observed increase in DD-endopeptidase activity, which preferentially targeted the deacetylated forms (Fig. 2c and d). Importantly, this increased PG hydrolytic activity was somewhat mitigated when *mreB* mutant cells were grown in the presence of excess Mg^2+^: the muropeptides resulting from DL-endopeptidase activity decreased to 9.3% of all muropeptides (Fig. 2d). Taken together, these results suggest that magnesium rescues deregulated DL-endopeptidase activity in *mreB* mutant cells.

Altogether, the increase in dl-endopeptidase and dd-endopeptidase activities, altered PG amidation, increased deacetylation and decreased dd-carboxypeptidase activity further indicate that homeostasis of PG hydrolysis is affected in the absence of *mreB*. Furthermore, magnesium may act –at least in part– through DL-endopeptidases to rescue the phenotype of *mreB* mutant cells.

### Deformation of *∆mreB* mutants is accompanied by anisotropic deregulation of sidewall synthesis/degradation

We next investigated the dynamics of PG synthesis and degradation in ∆*mreB* mutant cells using the non-toxic fluorescent d-amino-acid (FDAA) TAMRA d-lysine (TDL). In *B. subtilis* FDAAs are incorporated in the fifth position of the PG stem peptides^35^ through activity of transpeptidases^36^. Thus, TDL labelling allows visualization of transpeptidation reactions in vivo, i.e. in situ probing of newly inserted PG during growth^37^. Dilution of the TDL label in pulse-chase experiments can additionally inform about the pattern of PG modification/degradation^10^ as it results from both insertion of new PG and loss of the fluorescent d-amino-acid by the action of PG hydrolases.

We first visualized the sites of PG incorporation using conventional epifluorescence microscopy and structured illumination microscopy (SIM). Typical images are shown in Supplementary Fig. S4. In the presence of added Mg^2+^, the patterns of TDL staining of wild-type and *∆mreB* mutant cells were indistinguishable, as previously observed using fluorescently-labeled vancomycin as reporter of PG insertion^22,33,38^. TDL fluorescence was distributed in a regular, punctate pattern along the sidewalls and was dimmer at the cell poles, which are known to be inert regarding PG synthesis^39^ (Supplementary Fig. S4a,b). When no Mg^2+^ was added, the staining pattern of *∆mreB* cells was similar except that the cell poles were also stained (Supplementary Fig. S4c), also as previously observed using vancomycin staining^33,38^. Importantly, the sites of bulging and bending along the cell cylinder were also stained, indicating that PG is inserted along the sidewalls of ∆*mreB* cells regardless of their deformations. Bulged and bloated ∆*mreB* cells displayed a more intense and homogeneous staining (Supplementary Fig. S4c), suggestive of higher transpeptidase activity in these cells (see “Discussion”).

**Figure 3:**
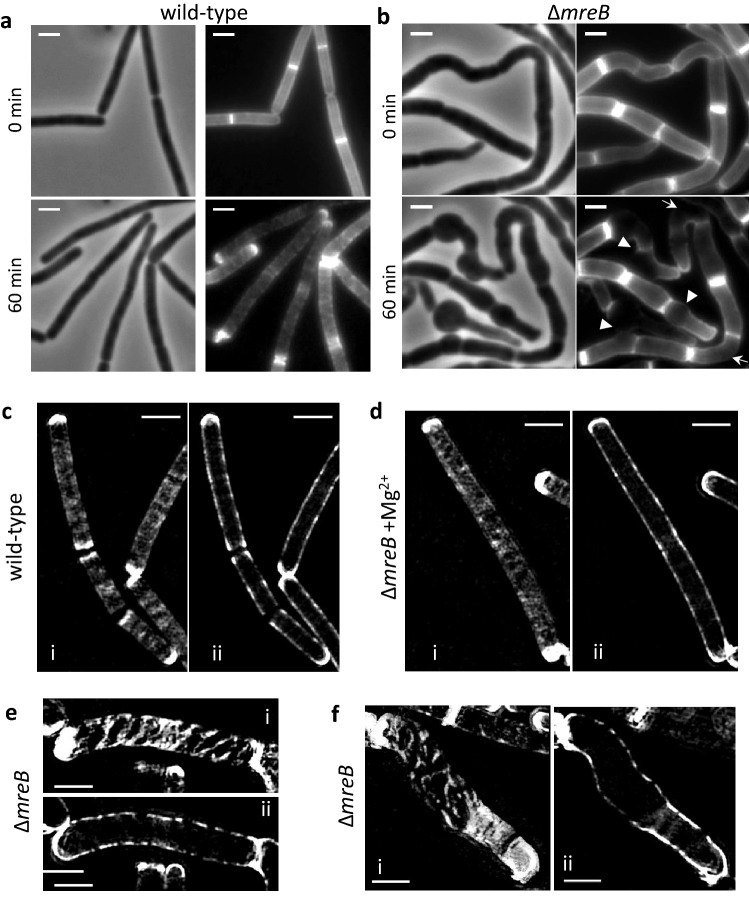
Peptidoglycan degradation along de sidewalls is anisotropic in Δ*mreB* cells. (**a**, **b**) TDL time-lapse pulse chase experiment in a microfluidics device. Wild-type (**a**) and Δ*mreB* (**b**) cells were grown in LB without added Mg^2+^, stained with TDL for 20 min, rinsed and further grown in LB. Images correspond to 0 min and 60 min after rinsing. Phase contrast (left) and epifluorescence (right) images are shown. Deformations of Δ*mreB* cells and the corresponding anisotropic loss of TDL label are indicated by white arrowheads (bulging cells) and arrows (bent cells). Scale bars, 2 µm. (**c**–**f**) SIM images of a similar TDL pulse-chase experiment performed in batch cultures. Wild-type (**c**) and Δ*mreB* cells growth in the presence (**d**) and in the absence (**e**, **f**) of added Mg^2+^. Cells were stained with TDL for 20 min, rinsed and allowed to grow for 35 min in fresh medium. SIM images of the cell top plane (i) and medial plane (ii) are shown. Scale bars, 2 µm.

We next investigated the dynamics of PG modification/degradation during sidewall elongation and deformation using TDL pulse-chase experiments. Exponentially growing cells were grown for one doubling time (~ 20 min) in the presence of TDL, rinsed and allowed to further grow in fresh medium. Disappearance of the fluorescence was first monitored in real time using microfluidics. In wild-type cells, after 60 min of growth, the TDL label was uniformly lost along the sidewalls and was retained at the cell poles, indicating that cells grew and divided during the experiment and that the cell poles are inert with respect to PG degradation/turnover too (Fig. 3a and Movie 2). In sharp contrast, in *∆mreB* cells large gaps without fluorescence were observed at the sites of bulging and at the longer outer face of bent cells (arrowheads and arrows, respectively, in Fig. 3b, Movie 1). We then imaged the pattern of dilution of TDL label at higher resolution by SIM. Cells were grown 20 min in the presence of TDL, washed and allowed to grow without the dye for two more generations. In both the wild-type (Fig. 3c) and the *∆mreB* mutant growing in the presence of excess Mg^2+^ (Fig. 3d), the staining over the cell surface became diffuse, with gaps in fluorescence intensity isotropically distributed along the sidewalls. In the absence of excess Mg^2+^, the *∆mreB* mutant showed a very different staining, with large gaps of fluorescence distributed in uneven patterns along the sidewalls (Fig. 3e and f). Bulging cells showed larger gaps in fluorescence at the sites of bulging, while the regions directly adjacent to the bulges and the poles remained very bright (Fig. 3f and Supplementary Fig. S5). Loss of TDL label can result from PG hydrolysis, dd-carboxypeptidase activity and/or PG synthesis. Since PG seemed homogeneously inserted in these regions (Supplementary Fig. S4c) and carboxypeptidase activity was decreased in ∆*mreB* cells (Supplementary Table S1), we concluded that PG hydrolase activity may be anisotropically deregulated.

### Inhibition of autolysins by magnesium is mediated by amidated PG and requires intact cells

**Figure 4:**
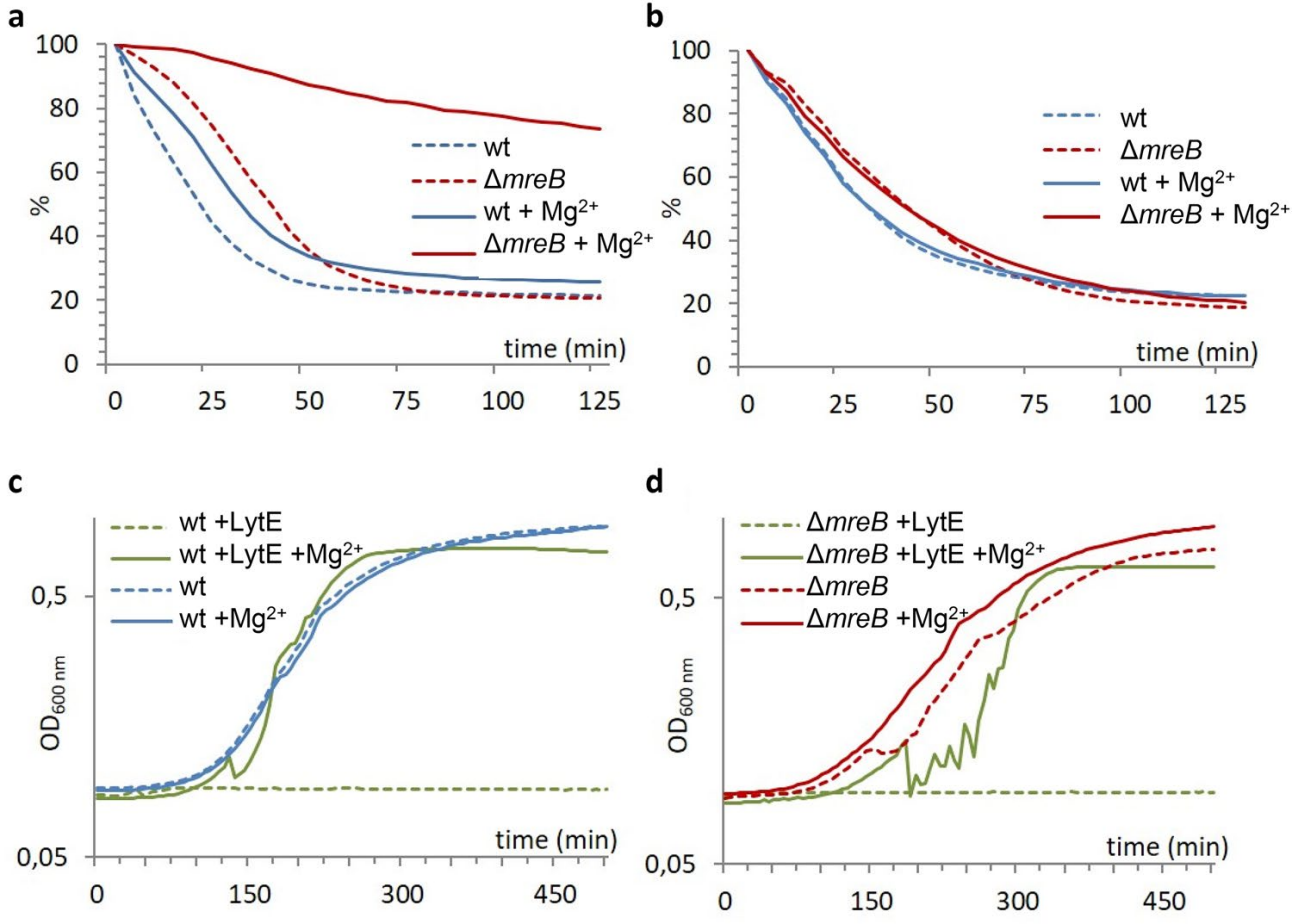
Magnesium inhibits the access of PG hydrolases to their substrate. (**a**, **b**) Autolysis curves of live cells (**a**) and of isolated sacculi (**b**) of the wild-type and the Δ*mreB* mutant grown to mid-exponential phase in LB medium containing 25 mM Mg^2+^ and autolysed in unsupplemented PBS (dashed lines) or in PBS containing 25 mM Mg^2+^ (plain lines). Autolysis rate is expressed as percentage of the maximum initial OD_600nm_. (**c**, **d**) Growth curve of the wild-type (**c**) and the Δ*mreB* mutant (**d**) strains in LB medium supplemented (plain lines) or not (dashed lines) with 25 mM Mg^2+^ in the presence or not of 100 µg/ml purified recombinant LytE.

We recently showed that in *B. subtilis* Mg^2+^ inhibits the lethal activity of some PG hydrolases^10^, which remain to be identified*.* The activity of a small subset of these (including the dl-endopeptidases LytE and LytF, the amidase LytC and the glucosaminidase LytD^4,40^) is enhanced by membrane depolarization, e.g. by membrane depolarizing agents such as sodium azide or by depolarisation of the membrane by a sudden shift from growth in rich medium to buffer such as PBS, and can be assayed in autolysis experiments. When excess Mg^2+^ is added to the autolysis medium, the autolysis rate of wild-type cells is reduced^10^ (Fig. 4a). Inhibition of autolytic activity increases with Mg^2+^ concentration, and the rate of cell autolysis is proportional to the apparent instantaneous growth rate of the cells^10^ (Supplementary Fig. S6a). To test the effect of mDAP amidation on Mg^2+^-mediated inhibition of autolysins, we compared the autolysis rate of wild-type cells grown in LB medium supplemented or not with Mg^2+^, i.e. containing ~ 29% and ~ 36% doubly amidated dimers, respectively (Fig. 2b and Supplementary Table S1), and lysed in the presence and in the absence of high Mg^2+^ in the autolysis buffer. The differential mDAP amidation had no effect on the autolysis rate when Mg^2+^ was not present in the autolysis buffer (Supplementary Fig. S6b). However, in the presence of Mg^2+^ cells displaying a higher degree of mDAP amidation autolysed more slowly (Supplementary Fig. S6b). Altogether, these data show that exogenous Mg^2+^ inhibits autolysins present in the CW in a growth-independent manner, and that this effect is mediated through doubly amidated dimers. mDAP amidation may in turn be modulated during growth to regulate PG hydrolytic activity, as previously suggested^10^. Addition of NaCl to the autolysis medium had the opposite effect than addition of MgSO_4_ and stimulated cell autolysis (Supplementary Fig. S6c), consistent with early reports that NaCl stimulates autolysis of intact *Staphylococcus aureus* cells^41,42^. When both NaCl and MgSO_4_ were added together to the autolysis buffer, the effect of one salt was balanced by the other (Supplementary Fig. S6c).

In order to further understand how Mg^2+^ ions inhibit PG hydrolases and rescue the phenotype of *mreB* mutant cells, we investigated the effect of excess Mg^2+^ on the autolysis rate of the ∆*mreB* mutant. The rate of autolysis of exponentially-growing ∆*mreB* cells was not appreciably different from the wild-type when no magnesium was present in the autolysis buffer (Fig. 4a). However, when excess Mg^2+^ was added to the autolysis medium, the autolysis rate of the Δ*mreB* mutant was dramatically reduced relative to the wild-type (Fig. 4a). The autolysis rate of native sacculi isolated from wild-type and ∆*mreB* cells was however unaffected by the presence of Mg^2+^ in the autolysis buffer (Fig. 4b). These results suggest that much of the inhibitory activity of Mg^2+^ on autolysins does not occur by direct interaction of Mg^2+^ with the enzymes but by another mechanism requiring intact cells, and that this mechanism may be more efficient in Δ*mreB* than in wild-type cells.

We next examined directly the effect of Mg^2+^ on the activity of two known PG hydrolases, by following the growth of cells in the presence of purified LytE or lysozyme. Addition of excess Mg^2+^ to the growth medium protected both the wild-type and the ∆*mreB* mutant against lysis by both exogenous LytE (Fig. 4c and d) and lysozyme (Supplementary Fig. S7a). Interestingly, addition of 25 mM Ca^2+^ to the growth medium had a similar effect as addition of 25 mM Mg^2+^ in protecting cells against lysis by lysozyme (Supplementary Fig. S7a), and in reducing autolysis of ∆*mreB* mutant cells (Supplementary Fig. S7b).

### Teichoic acids are not required for the inhibitory effect of magnesium on autolysins

Finally, we tested the possibility that the interaction of Mg^2+^ with phosphate groups of TAs (WTAs or LTAs) also affects the capacity of PG hydrolases to act on their substrate. We first tested a possible role of the membrane-embedded LTAs, which are thought to constitute a buffering zone scavenging and allowing control on divalent cations^43,44^. *B. subtilis* has three partially redundant LTA synthases (LtaS, YfnI and YqgS, believed to be the ‘house-keeping’, the ‘stress’ and a sporulation-specific LTA synthase, respectively)^45,46^. Autolysis of cells lacking *ltaS*, which are known to be affected in divalent cation homeostasis^46^, was inhibited by Mg^2+^to a similar extent as for wild-type cells (Supplementary Fig. S8a). This suggested that Mg^2+^-dependent inhibition of autolysins does not depend on the interaction of Mg^2+^ with LTAs. We then tested if it depends on the interaction of Mg^2+^ with WTAs. In the absence of TagO synthesis of WTAs is completely inhibited and *B. subtilis* cells become round^26^. The effect of Mg^2+^ on the autolysis of cells lacking WTAs could not be tested because the Δ*tagO* null mutant did not grow in liquid LB without excess Mg^2+^ (Supplementary Fig. S9a), and Δ*tagO* cells clump severely even in the presence of Mg^2+^^26^, making autolysis readings erratic. We therefore investigated the effect of Mg^2+^ on the growth and the morphology of a *tagO* conditional mutant. TagO-depleted cells required the presence of excess Mg^2+^ to grow at the wild-type level (Supplementary Fig. S9a). Furthermore, deformation of the cells was rescued by Mg^2+^ (Supplementary Fig. S9c,d). In agreement with these results, an early report indicated that Mg^2+^ reversed the altered cell morphology of *rodC* mutants, bearing a mutation in the major WTA polymerizing enzyme gene, *tagF*^47^. Taken together, these results suggested that the rescue role of Mg^2+^ does not depend on its interaction with WTAs.

## Discussion

In *B. subtilis,* depleting MreB leads to cell deformation and eventually lysis that are rescued when Mg^2+^ is present at millimolar concentrations in the growth medium^22^. Our AFM and TEM analyses revealed that in presence of 25 mM Mg^2+^ the structure and mechanical properties of the CW of *∆mreB* cells are indistinguishable from wild-type cells. However, when no excess Mg^2+^ was present *∆mreB* cells displayed an irregular, rough cell surface with decreased stiffness, indicating that cell deformation results from altered CW homeostasis. In agreement with this, muropeptides analysis showed that the activities of two types of PG hydrolases, namely endopeptidases (DL- and DD-endopeptidases) and dd-carboxypeptidases, are deregulated (upregulated and downregulated, respectively), and that amidation and deacetylation, two PG modifications known to modulate PG hydrolases^8,10^, are also deregulated. TDL pulse-chase experiments also suggested that the activity of PG hydrolases is increased along the sidewalls of *∆mreB* cells. The dd-carboxypeptidase PBP5, which removes the C-terminal d-Ala residue from the pentapeptide chain of the PG precursor, has been shown to contribute to the loss of fluorescent d-amino acid labeling in *B. subtilis*^37^. dd-carboxypeptidase activity was however reduced in *mreB* mutant cells (Supplementary Fig. S3 and Table S1), and thus could hardly explain an increased loss of TDL labelling. Furthermore, the activity of PBP5 does not affect the growth or the morphology of *B. subtilis*^48^. Thus, while spatial dysregulation of PBP5 could explain the anisotropic loss of TDL label in *∆mreB* cells, such dysregulation could not explain their bulging and lysing phenotype. We conclude that this phenotype is most likely due to elevated DL-endopeptidase activity over the sidewalls. Although the activity of DD-endopeptidases was also increased in the *mreB* mutant, this increase was not mitigated by the presence of excess magnesium, while the increase in activity of DL-endopeptidases was largely compensated.

Higher PG hydrolytic activity could produce partially degraded PG consistent with the rough ‘peeling’ surface of *∆mreB* cells observed by TEM and AFM, and explain the lytic phenotype of the mutant. Importantly, in *B. subtilis* MreB isoforms have been shown to regulate the activity of the co-essential dl-endopeptidases LytE and CwlO by mechanisms that remain unknown^17^. Mbl is crucial for CwlO function and, accordingly, it is synthetically lethal with LytE^17^. In contrast, MreB and MreBH appeared more important for LytE function but are not synthetically lethal with CwlO, possibly because of functional overlap^17^. Furthermore, the localization of LytE to the lateral CW was shown to depend on *mreBH* in cells growing in the presence of excess Mg^2+^^18^. It would be therefore tempting to think that the phenotype of *∆mreB* cells depends on uncontrolled activity of LytE over the sidewalls, which is inhibited by excess Mg^2+^. However, a previously reported *∆mreB ∆lytE* double mutant still displayed extensive lysis^17^, suggesting that other PG hydrolases are also at play. Further studies will be required to identify what enzymes, among the > 40 putative PG hydrolases encoded by the of *B. subtilis* genome^4^ (including > 12 putative endopeptidases, 7 of which contain DL-endopeptidase domains), are dysregulated in the absence of MreB. MreB might control their activity, levels or spatial organization in the cell, or a combination of these.

It was recently shown that bi‐functional (Class A) penicillin‐binding proteins (aPBPs) polymerize PG outside the MreB-associated Rod complexes, inserting material in a diffuse manner along the sidewall. Both systems play major roles in PG synthesis but are partially interdependent and thought to collaborate with each other at some level^49^. The proposed model is that the Rod system may build the primary PG structure inserting material in a circumferential manner while aPBPs may add to it and/or fill in gaps arising during sidewall expansion^49,50^. Intriguingly, inactivation of PBP1, the major vegetative aPBP, was reported to restore the viability and to suppress the bulging and lysing phenotype of *B. subtilis ∆mreB* cells^51^. These findings and our finding that bulging and lysis of the *mreB* mutant result from dysregulated PG hydrolases can be reconciled by the hypothesis that MreB regulates one or more PG hydrolases that are associated to PBP1 activity. Interestingly, endopeptidases were recently shown to specifically activate PG synthesis by aPBPs in *E. coli*^52^. Thus, one attractive possibility is that MreB may directly or indirectly control endopeptidases that are coupled to the insertion of PG by PBP1. In the absence of *mreB*, increased endopeptidase activity would stimulate PBP1 activity, leading to cell bulging and rounding (resulting from PBP1-dependent diffuse insertion of PG). In this scenario, both absence of PBP1 and inhibition of upregulated PG hydrolases by excess Mg^2+^ would prevent cell bulging of *∆mreB* cells, as observed.

Mg^2+^ has been shown to rescue *mreB* mutants but also other CW-related mutants including *mbl, mreC, mreD* and *ponA* (encoding PBP1)^21^. Moreover, *B. subtilis* cells deform when growing in minimal media with low Mg^2+^ concentration^51^. The exact mechanism by which Mg^2+^ rescues viability and/or cell shape remains unknown but we recently showed that it inhibits PG hydrolases and reduces amidation of dimeric muropeptides^10^. CW modifications are of particular importance for the control of PG hydrolases. Most PG hydrolases are proteins with a high pI, positively charged at neutral pH, and it has been proposed that they interact electrostatically with the anionic phosphate backbone of TAs. The modification of TAs by addition of cationic D-alanyl esters (D-alanylation) is therefore thought to reduce the activity of PG hydrolases by decreasing their binding capacity, by charge compensation^2^. However, mDAP amidation seems to enhance the activity of autolysins instead, despite neutralizing one carboxyl group of PG. Interestingly, doubly amidated dimers were the only detected substrate of DL-endopeptidases in our UPLC analysis (Fig. 2e), and we found that they mediate the inhibition of autolytic activity by Mg^2+^ (Supplementary Fig. S6a).

Mg^2+^ could regulate the activity of PG hydrolases by interacting with them directly or by affecting their capacity to interact with the CW. In the latter case, Mg^2+^ could compete with PG hydrolases to bind negatively charged groups in the CW. Alternatively, Mg^2+^ ions could induce changes in CW organization (by crosslinking negatively charged groups) that could modify the interaction of PG hydrolases with their substrate or their mobility in the sacculus. Our finding that autolysis of isolated native cell walls was much less inhibited by excess Mg^2+^ than autolysis of intact cells argues against direct inhibition of PG hydrolases by Mg^2+^. Our XPS data are consistent with previous findings that metal cations bind to both phosphate groups of TAs and carboxyl groups of PG peptides^29,31^. However, our results suggest that Mg^2+^-dependent inhibition of PG hydrolases does not depend on the interaction of Mg^2+^ with TAs. Furthermore, Mg^2+^ has been shown to inhibit autolysis also in exponentially growing *E. coli* cells^53,54^ and other unrelated Gram-negative bacteria^55,56^, which lack TAs. We conclude that the underlying primary mechanism is independent of TAs.

In our autolysis experiments, addition of excess Mg^2+^ resulted in the reduction of autolysis rate and protected cells against exogenously added CW degrading enzymes such as recombinant LytE and lysozyme. Importantly, this effect was not specific to Mg^2+^. Addition of excess Ca^2+^ to the growth medium at the same concentration (25 mM) had a similar effect. Furthermore, 25 mM Ca^2+^ was able to maintain the rod shape of ∆*mreB* almost as efficiently as Mg^2+^ (Movie 3). CW metal binding data previously showed that the metal binding characteristics of Mg^2+^ and Ca^2+^ are similar in *B. subtilis*^30^. Interestingly, both Mg^2+^ and Ca^2+^ reduce the rate of autolysis in *E. coli* too^53^, further suggesting that the effect of Mg^2+^ has an important electrostatic component.

In summary, this work demonstrates that deformation of *∆mreB* mutant cells is mainly due to unbalanced PG synthesis and degradation. Mg^2+^ restores this equilibrium and maintains rod shape by inhibiting the activity of PG hydrolases. Our results also suggest that Mg^2+^ may inhibit PG hydrolases by affecting their capacity to act on their substrate. Further investigations will be required to confirm this hypothesis and to elucidate which PG hydrolases exactly are dysregulated in the absence of *mreB* and how. Another important question that remains to be answered is which PG hydrolases are associated to the PG synthetic activity of the Rod and the aPBPs systems, and how they are regulated.

## Methods

### Bacterial strains and growth conditions

The strains used in this study are shown in Supplementary Table S3. Kanamycin was used at 10 µg/ml and erythromycin at 1 μg/ml. Lysozyme was purchased from Sigma. Strains were grown at 37 °C under continuous shaking in LB medium supplemented or not with MgSO_4_ or CaCl_2_ as indicated in the text. Growth curves were performed in 96-well plates in a Synergy microplate reader (Biotek). Overnight cultures, inoculated from an isolated colony, were diluted to an OD_600_ of 0.001 and incubated at 37 °C with continuous agitation. For microscopy experiments, overnight cultures were diluted at least × 1000 in fresh medium and allowed to grow to early exponential phase under the conditions indicated in the text.

### Atomic Force Microscopy (AFM)

Overnight cultures were diluted into fresh LB (containing or not 25 mM MgSO_4_) and grown at 37 °C. When cells reached OD_600nm_ ~ 0.4, 1 ml of culture was pelleted by centrifugation, rinsed twice and allowed to settle for 20 min on a piranha cleaned, Cell-Tak-modified (Corning), glass bottom Petri dish (WPI, Meyer et al. 2010). Unattached cells were washed away and the attached cells covered with culture medium for further analysis with AFM. Imaging and force measurement were realized using a Nanowizard 3 Atomic Force Microscope (JPK Instruments AG, Berlin, Germany) coupled to a Zeiss inverted microscope. Topographic images and elasticity cartography were generated simultaneously using the Quantitative Imaging® (QI) mode with DNP cantilevers (0.06–0.24 N/m, Bruker) in culture medium. The ramp size and speed were set at 400 µm and 25 µm/s respectively and the maximum load was 6 nN, maps of 128 by 128 force curves were realized. Calibration of the probe was carried out by measuring the deflection sensitivity on glass surface and spring constant was determined using the thermal tune method. Young’s moduli were calculated by fitting force curves with the Sneddon model using the JPK software.

### Transmission electron microscopy (TEM)

For transmission electron microscopy (TEM), exponentially growing cells were harvested by gentle centrifugation, washed 2 times with PBS 1x, and fixed with 2% glutaraldehyde in 0.1 M sodium cacodylate buffer pH 7.2 for 2 h at room temperature. Cells were then contrasted with Oolong Tea Extract (OTE) 0.5% in cacodylate buffer and post-fixed with 1% osmium tetroxide containing 1.5% potassium cyanoferrate. Samples were gradually dehydrated in ethanol (30% to 100%), substituted progressively in a mix of propylene oxyde-Epon and embedded in Epon (Delta microscopies – Labège France). Thin sections (70 nm) were collected onto 200 mesh copper grids, and counterstained with lead citrate. Grids were examined using a Hitachi HT7700 electron microscope operated at 80 kV and images were acquired with a charge-coupled device camera (AMT). TEM was performed in the Microscopy and Imaging Platform MIMA2, INRAE Jouy-en-Josas, France.

### X-ray Photoelectron spectroscopy (XPS)

The analysis of solid surfaces by XPS is based on irradiation with an X-ray beam and emission of electrons, the kinetic energy of which is analysed. This provides, after adequate calibration, a spectrum plotted as a function of the binding energy (in eV) of the emitted photoelectrons. Owing to inelastic scattering of electrons in a solid, the information collected concerns the outermost molecular layers at the surface, typically a thickness of 3 to 10 nm. For XPS analysis, exponentially growing cultures were harvested by centrifugation. Pellets were rinsed three times with ultrapure water, quickly frozen in liquid nitrogen and stored at − 80 °C. Cell powder was placed in a stainless steel trough with an inner diameter of 4 mm, and mildly pressed with a polyacetal cylinder cleaned with isopropanol to obtain a smooth surface. Analyses were realised on two sets of independent culture. XPS analyses were performed using an SSI X-probe (SSX- 100/206) photoelectron spectrometer from Surface Science Instruments (USA). The flood gun energy was set at 8 eV. The analyzed spot has an elliptical shape and a surface area of 1.4 mm^2^. The spectra were recorded using the following sequence: survey, C 1s together with K 2p, O 1s, N 1s, P 2p, Ca 2p, Mg 2p, S 2p, Na 1s, Cl 2p and finally C1s again to check for the absence of sample degradation.

The C–(C,H) component of the C1s peak of carbon was fixed to 284.8 eV to set the binding energy scale. Data treatment was performed with the CasaXPS program (Casa Software Ltd, UK), and some spectra were decomposed with the least squares fitting routine provided by the software with a Gaussian/Lorentzian (85/15) product function and after subtraction of a nonlinear baseline (Shirley). Molar fractions were calculated using peak areas normalized based on acquisition parameters and sensitivity factors provided by the manufacturer. To determine the surface concentration of charges, positive charges were computed as follows: C_Na_ + C_K_ + 2·C_Ca_ + 2·C_Mg_ + C_Nprot_, where C stands for the concentration determined for each element and Nprot stands for protonated amines, while negative charges are equal to C_P_ since phosphorus is attributed to TA (with one negative charge per P atom).

### Muropeptides analysis by UPLC and mass spectrometry

*B. subtilis* wild-type and ∆*mreB* mutant strains were grown in 1 L cultures in LB medium (containing or not 25 mM MgSO_4_) to OD_600nm_ 0.5. Peptidoglycan purification, digestion with mutanolysin and UPLC and mass spectrometry were performed as described previously^10^. The peaks identified by MALDI-TOF were integrated using the Agilent software to estimate the amount of each muropeptide. The reported crosslinking index (%) was calculated as described in^57^: % dimers/(% monomers + 2x % dimers). % of DL-endopeptidase products = ds2 + ds2-Ac + ds3a2a + ds3a2a-Ac. % of DD-endopeptidase products = ds3 + ds3a + ds3a-Ac. ds refers to disaccharide (GlcNAc-MurNAc). The number indicates the length of the stem peptide: 2, dipeptide (L-Ala-D-iGlu); 3, tripeptide (L-Ala-D-iGlu-mDAP); 4, tetrapeptide (L-Ala-D-iGlu-mDAP-D-Ala); 5, pentapeptide (L-Ala-D-iGlu-mDAP-D-Ala-D-Ala). (a), amidated; (-Ac), missing an acetyl group.

### Labeling with TDL and microscopy

For TDL labeling, 100 µl of culture in early exponential phase was stained with 1 mM TDL (TAMRA Red d-lysine) at 37 °C for 2 min (PG insertion labeling experiments) or 20 min (PG degradation pulse-chase experiments). Cells were rinsed and imaged using a Zeiss Elyra PS1 microscope (3D-SIM mode) or an inverted Nikon microscope (Eclipse Ti-E) (phase contrast and epifluorescence microscopy) as described previously^10^. Microfluidics experiments were performed using the CellASIC ONIX (Millipore) microfluidics system also as described in^10^. For growth experiments, images were taken every 20 s. For TDL pulse-chase experiments, phase contrast and fluorescence images were taken every 5 min.

### Measurement of autolysis

Overnight liquid cultures of *B. subtilis* were diluted × 2000 times in LB containing or not 25 mM MgSO_4_ and incubated with continuous agitation at 37˚C until mid-exponential phase (OD_600nm_ = 0.4–0.6). For whole cells autolysis experiments, either cultures were washed 3 times in PBS and then resuspended in PBS (containing or not 25 mM MgSO_4_), or 75 mM sodium azide was added to the growing culture. For isolated native cell wall autolysis experiments, cells were harvested by centrifugation, washed with PBS and then sonicated in ice several times, until most of the cells were broken. Intact cells were removed by centrifugation at 2 000 × g for 5 min. The supernatant containing isolated cell walls was then washed 3 times in PBS and resuspended in PBS containing or not 25 mM MgSO_4_. The OD_600nm_ was monitored every 5 min to follow the rate of cell lysis.

### Production and purification of LytE

The *lytE* gene from *B. subtilis* 168 wild-type strain was codon optimized for optimal expression in *E. coli*. The resulting gene was synthesized (along with a hexahistidine tag at its N-terminus) by Dna2.0 and cloned into pD861-SR (Dna2.0), giving the plasmid pLytE. For protein expression, *E. coli* BL21 cells were transformed with pLytE (strain eRCL007) and grown in LB medium at 37 °C. Expression was induced at mid-exponential phase with 0.2% rhamnose and cells where further grown at 37 °C for 4 h. Cells were collected by centrifugation and frozen as pellets until purification. Protein purification was carried out as described in^58^**.**

### Significance statement

In bacteria, the peptidoglycan cell wall is the major determinant of cell shape and the main target of antibiotics. In rod-shaped bacteria, actin-like MreB proteins shape the cylindrical cell wall by orienting the movement of peptidoglycan-synthesizing enzymes. Here, we use a combination of biophysical, biochemical and cell biology approaches in the model bacterium *Bacillus subtilis* to address the mechanisms underlying two longstanding questions in the field: the bulging and lysis phenotype of *mreB* mutants, and the mysterious rescuing role of this phenotype by millimolar concentrations of magnesium. We show that the morphological defects of *mreB* mutants are due to dysregulated peptidoglycan-hydrolyzing enzymes, and that magnesium restores viability and rod shape by inhibiting the activity of these enzymes.

## Supplementary Information

Supplementary Video 1.

Supplementary Video 2.

Supplementary Video 3.

Supplementary Figures and Tables.
