# Supplementary material for: Magnesium rescues the morphology of Bacillus subtilis mreB mutants through its inhibitory effect on peptidoglycan hydrolases
Source: Sci Rep. 2022 Jan 21;12:1137. doi: 10.1038/s41598-021-04294-5 (PMC8782873; doi:10.1038/s41598-021-04294-5)
Supplement: Supplementary file 4 — Supplementary Figures and Tables. [file 41598_2021_4294_MOESM4_ESM.pdf]

Supplementary Information

for

Magnesium rescues the morphology of *Bacillus subtilis mreB* mutants through its inhibitory effect on peptidoglycan hydrolases

Benoit Tesson[a*], Alex Dajkovic[a,d], Ruth Keary[a], Christian Marlière[b], Christine C. Dupont-Gillain[c] and Rut Carballido-López[a*]

[a] Micalis Institute, INRAE, AgroParisTech, Université Paris-Saclay, 78350 Jouy-en-Josas, France.

[b] Laboratoire de Physique des Solides, LPS, University Paris-Saclay, CNRS, Orsay, France.

[c] Université catholique de Louvain, Institute of Condensed Matter and Nanosciences, Bio- and Soft Matter division, 1348, Louvain-la-Neuve, Belgium.

[d] Present address: Biomillenia (Design Pharmaceuticals), BIOCITECH, 93230 Romainville, France.

[*] To whom correspondence should be addressed.

Email: tessonben@gmail.com and rut.carballido-lopez@inrae.fr

Supplementary Tables S1 to S3

Supplementary Figures S1 to S9

Legends for Movies 1 to 3

References

Supplementary Tables

Table S1. Quantification of different muropeptides eluted in the UPLC runs shown in Fig. S3 (peak numbers refer to Figure S3). DD-endopeptidase products are in green; DL-endopeptidase products are shown in blue; the dominant peak of the dimer containing singly amidated mDAP is highlighted in red. The percentage of each peak was calculated as the ratio of the peak area over the sum of areas of all peaks identified in the corresponding chromatogram. ds refers to disaccharide (GlcNAc-MurNAc) and is followed by numbers which indicate the peptide length, as follows: 2 = dipeptide (L-Ala-D-iGlu), 3 = tripeptide (L-Ala-D-iGlu-mDAP), 4 = tetrapeptide (L-Ala-D-iGlu-mDAP-D-Ala), 5 = pentapeptide (L-Ala-D-iGlu-mDAP-D-Ala-D-Ala). a = amidated. –Ac = deacetylated. Anhydro = anhydromuropeptide.

Peak nr.	Proposed structure	% of all peaks			
		wt	wt + Mg^{2+}	*ΔmreB*	*ΔmreB* + Mg^{2+}
1	**ds3**	2,9	4,09	3,07	3,52
2	**ds3a**	17,78	18,79	21,14	20,73
3	**ds3a-Ac**	1,81	1,68	3,31	3,25
4	**ds2**	3,24	3,08	4,53	3,53
5	**ds3a4**	0,86	0,75	1,25	1,05
6	**ds2-Ac**	1,12	1,65	1,16	1,43
7	**ds3a2a**	0,86	0,75	1,46	1,24
8	**ds3a2a-Ac**	2,89	2,14	4,84	3,14
9	**ds3a2a, ds3a3, ds3a4a**	1,6	1,02	2,46	1,353
10	**ds5a**	1,01	0,61	3,37	1,61
11	**ds3 anhydro, ds3ads4-GlcNAc**	1,19	1,33	0,85	1,04
12	**ds3ds4, ds44**	0,94	1,43	0,72	0,85
13	**ds3ads4**	12,27	20,81	6,66	10,65
14	**ds3ads4, ds4a4**	3,36	4,35	3,1	4,32
15	**ds3ads4a-GlcNAc, ds3ads4-Ac, ds3a anhydro**	5,25	4,59	3,57	4,28
16	**ds3ads4a**	32,49	25,56	29,02	28,79
17	**ds3ads4a, ds3ads4a-Ac**	3,52	3,04	4,18	4,64
18	**ds3ads4-Ac**	4,67	2,73	5,31	4,57
	crosslinking index	0,42	0,41	0,39	0,40

Table S2. Positive and negative charges detected by XPS in the wild–type and the Δ*mreB* and the Δ*tagO* mutants grown in LB medium supplemented or not with 25 mM Mg^{2+}. Results of two independent sets of experiments, expressed as molar fractions (in %), excluding hydrogen. Positive charges (charges +) = [bound metals (Na+2·Ca+K+2·Mg) + protonated amines (Nprot)]. Negative charges (charges –) come from phosphate groups (P). The concentration of each element is indicated in Table 1.

	Nprot	metals	**Charges +**	**Charges -**
**wt**	0,4	2,1	2,5	2,4
	0,4	2,5	2,9	2,2
**wt +Mg^{2+}**	0,2	3,2	3,4	1,7
	0,5	2,9	3,4	2,0
**Δ*mreB***	0,5	3,3	3,8	2,4
	0,6	2,0	2,6	2,0
**Δ*mreB* +Mg^{2+}**	0,5	3,4	3,9	2,9
	0,5	2,4	2,9	2,0
**Δ*tagO* +Mg^{2+}**	1,0	2,1	3,1	0,2
	1,1	2,9	4,0	0,2

Table S3. List of strains used in this study

Strain name	Genotype	Reference
168	*trpC2*	[1]
3725	*trpC2 ΔmreB* (Kan)	[2]
EB1451	*hisA1 argC4 metC3* Δ*tagO* (Erm)	[3]
NC229	*trpC2 ΔtagO* (Ery) *amyE*::$P_{hyperspank}$-*tagO* (Kan)	[4]
BKK07710	*trpC2* Δ*ltaS* (Kan)	Bacillus Genetic Stock Centre
eRCL007	6xHis-LytE overproduction strain	This study

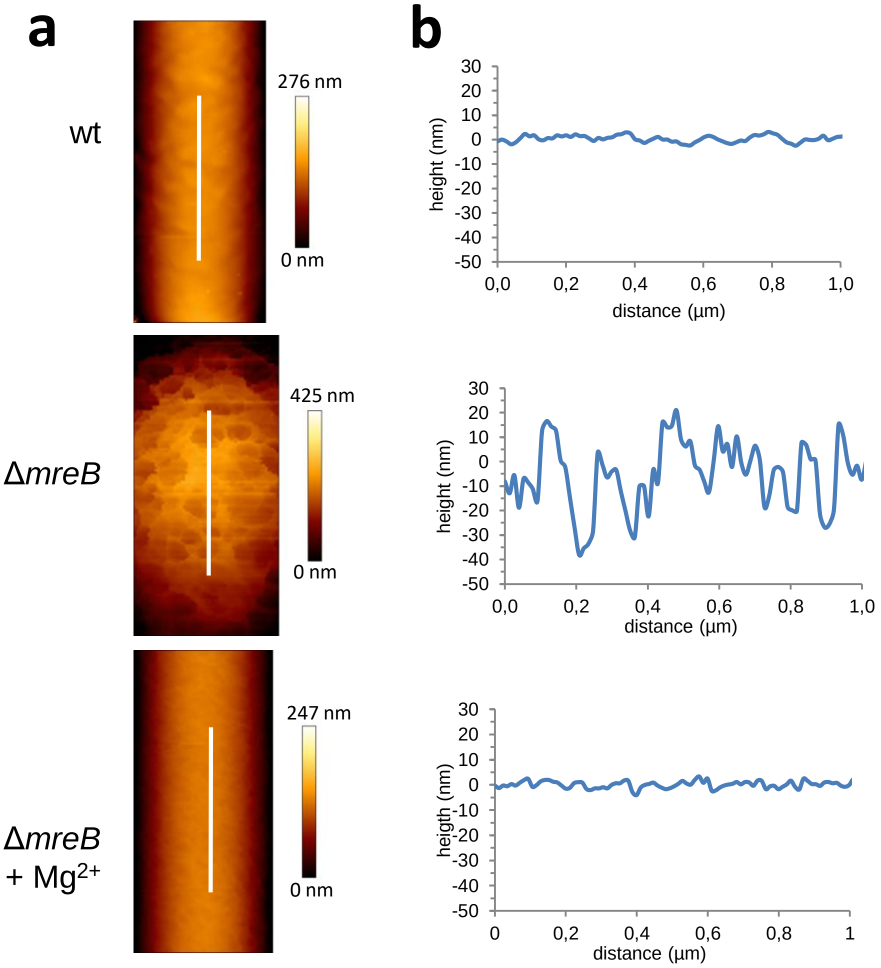

Figure S1. Heigth profiles (b) from longitudinal transects (white lines) in the AFM topography maps (a)

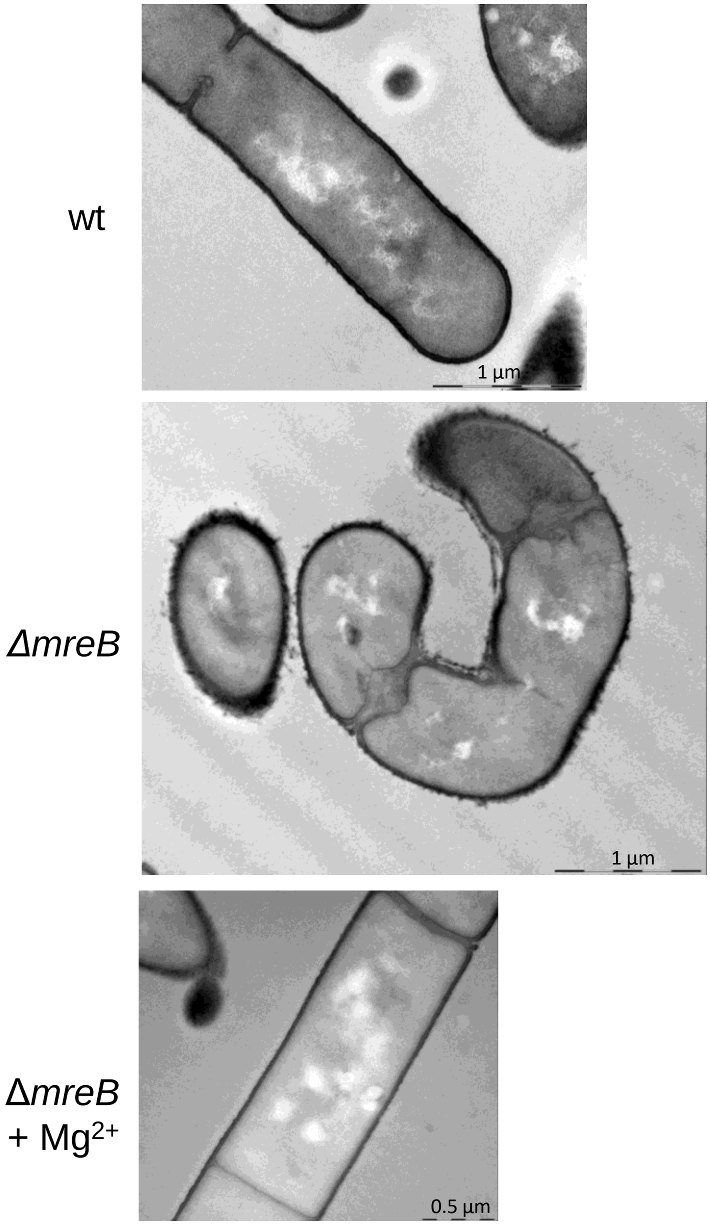

Figure S2. Transmission electron microscopy micrographs of representative transversal sections of vegetative wild-type and Δ*mreB* mutant cells grown in LB medium in the presence and in the absence of 25 mM Mg^{2+}.

Tesson *et al.*
Figure S3

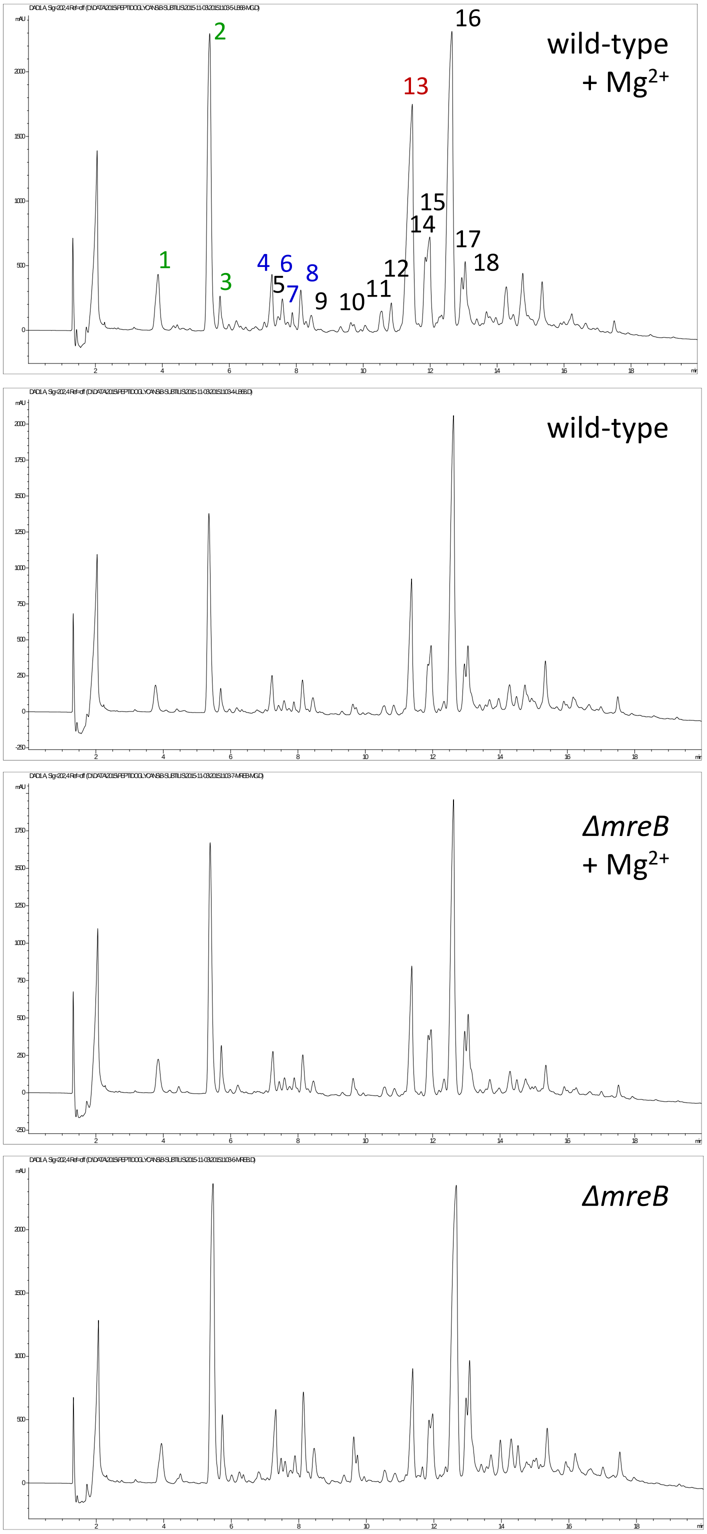

Figure S3. UPLC profiles of muropeptides of PG purified from the wild-type and the *ΔmreB* mutant cells growing exponentially in LB medium in the presence and in the absence of 25 mM Mg^{2+}. Peaks were labelled from 1 to 18 following their retention time. Their structure is shown in Table S1. Peaks labelled in red correspond to amidated dimeric species. Peaks labelled in green correspond to DD-endopeptidase products; peaks labelled in blue correspond to DL-endopeptidase products, The dominant peak of the dimer containing singly amidated mDAP is highlighted in red.

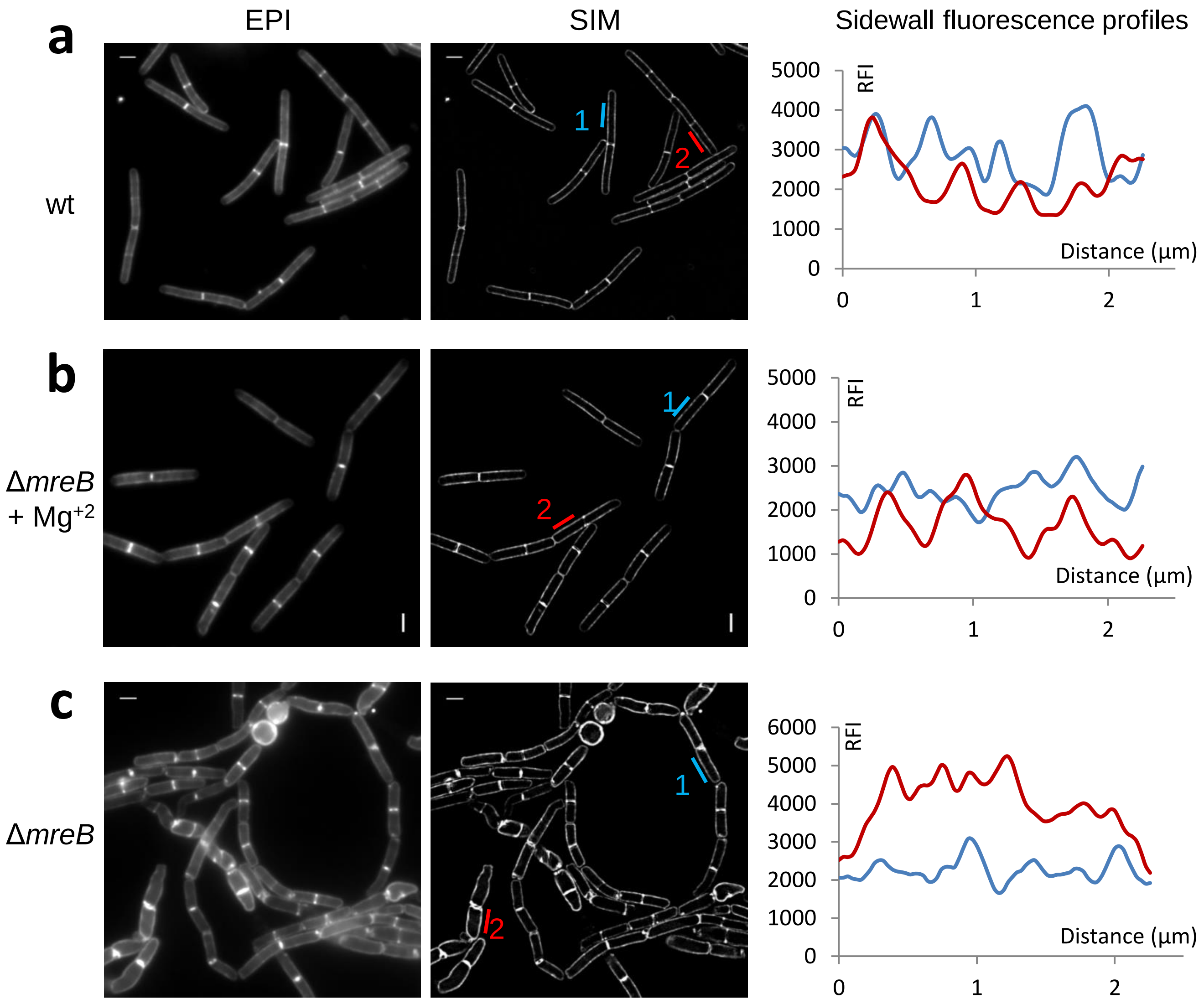

Figure S4. Dynamics of PG synthesis**.** Wild-type (a) and *ΔmreB* cells grown in the presence (b) and in the absence of added Mg^{+2} (c) stained for 2 min with 1 mM TDL. Epifluorescence (EPI) and SIM images, and fluorescence profiles along the sidewalls of the cells labelled 1 (blue) and 2 (red) in the SIM images. Scale bars, 2 µm.

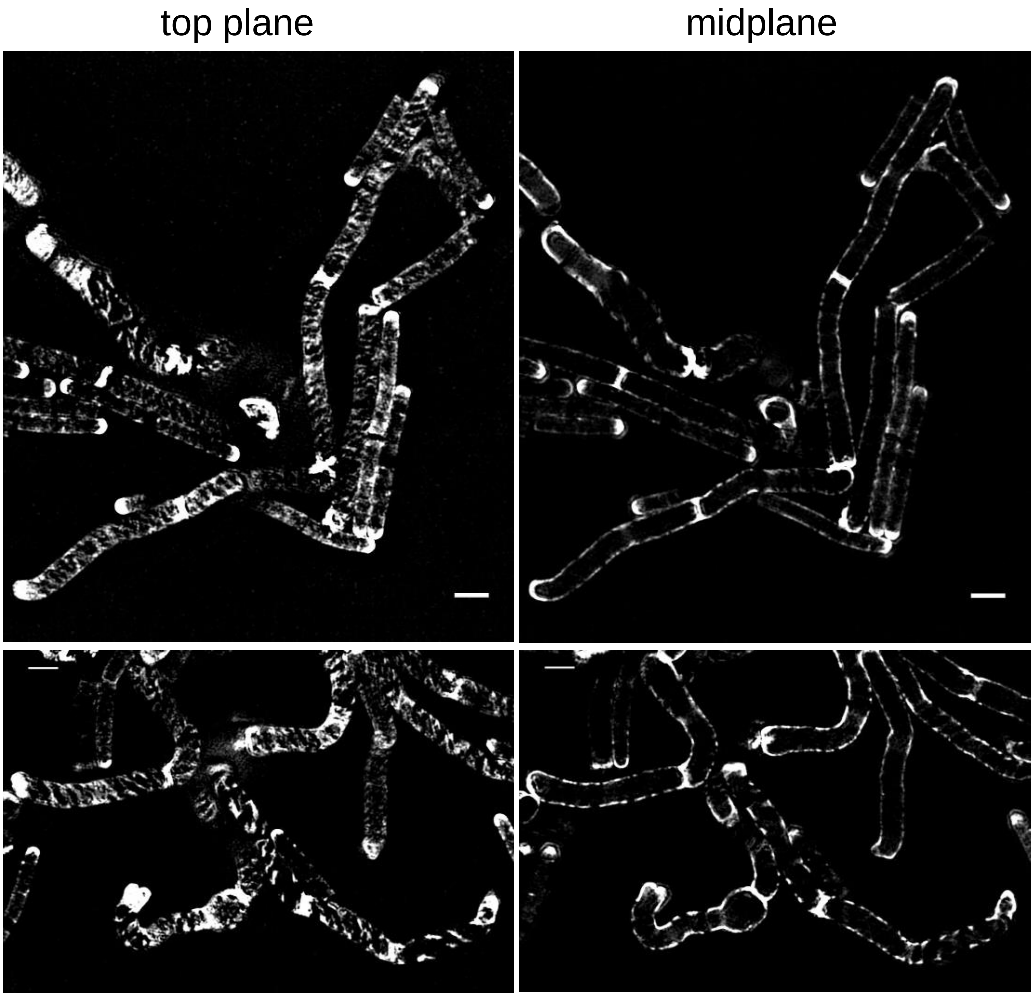

Figure S5. SIM images of the TDL pulse chase experiment performed in batch cultures of *Δ*mreB strain grown in LB medium without added Mg^{+2}, stained 20 min with TDL, rinsed and allowed to grow for 35 min in fresh LB. Scale bars, 2 µm.

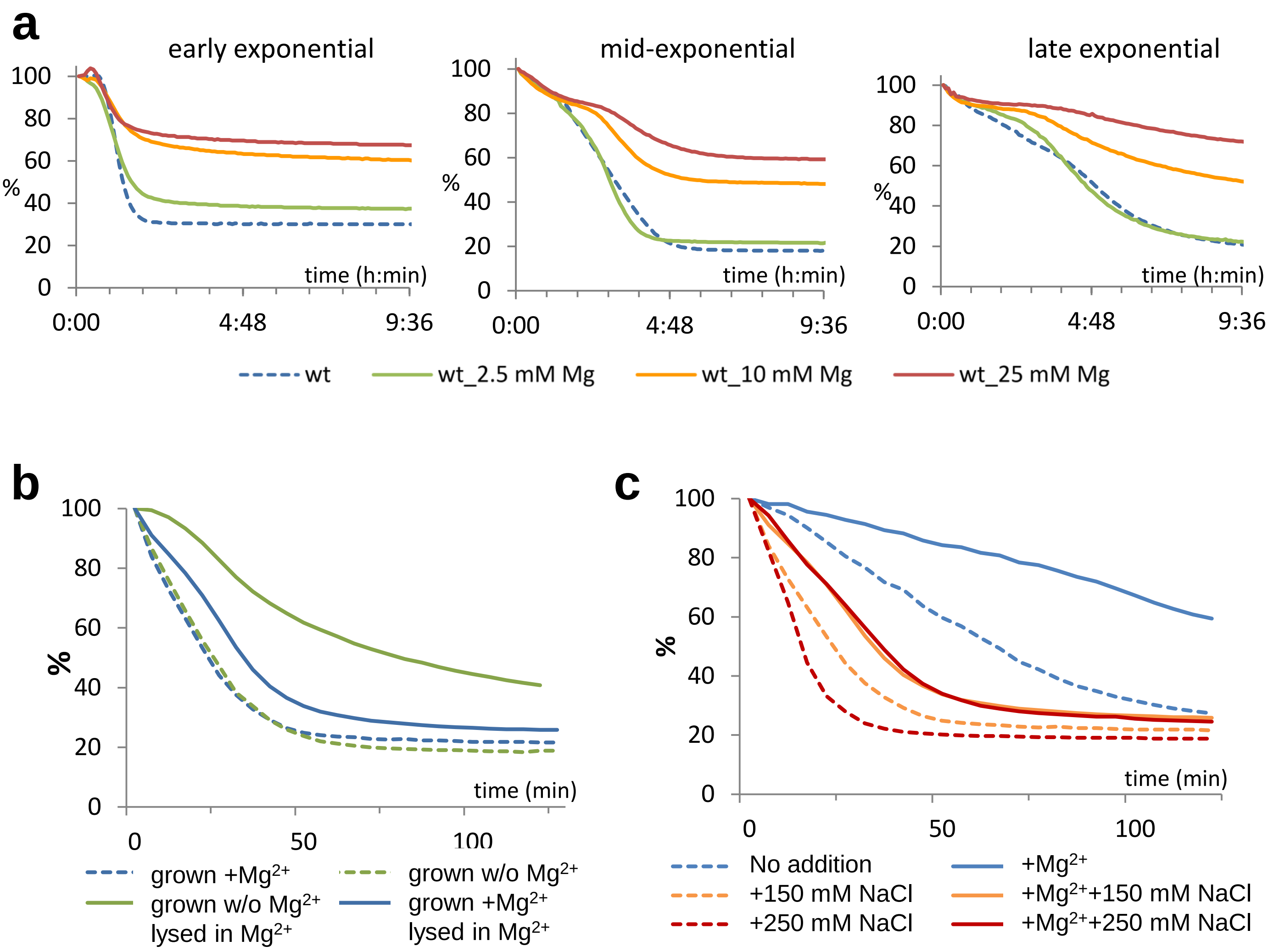

Figure S6. Autolysis curves of wild-type live cells in the presence of divalent cations. (**a**) Cells were grown to early exponential (OD_{600} 0.2-0.3), mid-exponential ($0D_{600}$ 0.5-0.6) and late exponential (OD_{600} 0.7-0.8) in LB medium supplemented with different concentrations of Mg^{+2} and autolysed by 75 mM sodium azide. (**b)** Cells were grown to mid-exponential phase in the presence (blue lanes) or in the absence (green lanes) of 25 mM Mg^{+2} and autolysed in PBS in the presence (plein lanes) and in the absence (dashed lines) of 25 mM Mg^{+2}. (**c)** Cells were grown in the presence of 25 mM Mg^{+2} and autolysed in PBS supplemented with 150 mM or 250 mM NaCl with or without 25 mM Mg^{+2}. Autolysis curves are expressed as a percentage of the maximum initial OD_{600}.

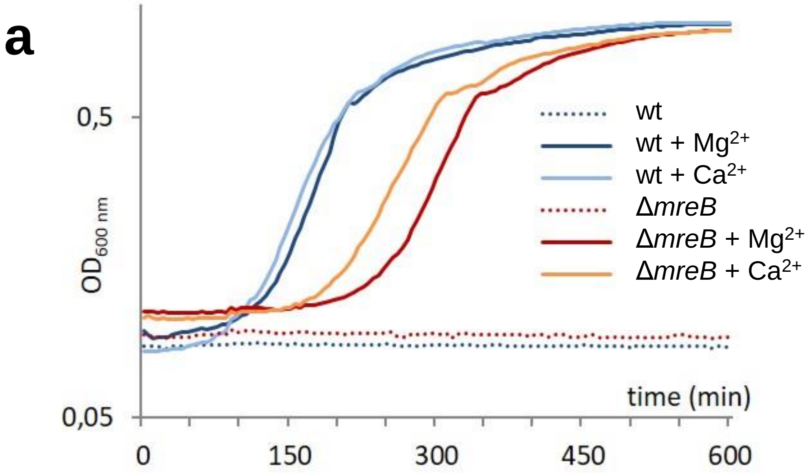

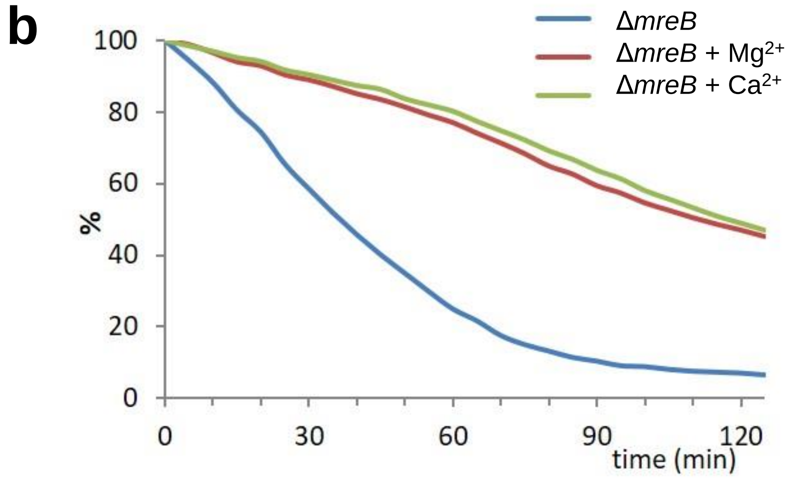

Figure S7. (**a**) Growth curve of the wild-type and the *ΔmreB* strain in the presence of 0.1 mg/ml of lysozyme in LB medium with or without addition of 25 mM $MgSO_4$ or 25 mM $CaCl_2$, as indicated. (**b)** Autolysis curve of the *ΔmreB* mutant grown in LB medium in the presence of 25 mM Mg^{+2} and autolysed in LB supplemented with 75 mM azide with or without addition of 25 mM $MgSO_4$ or 25 mM $CaCl_2$. Autolysis experiments in the presence of Ca^{2+} could not be done by resuspension in PBS because $CaCl_2$ is not soluble in PBS. Autolysis curves are expressed as percentage of maximum $OD_{600.}$

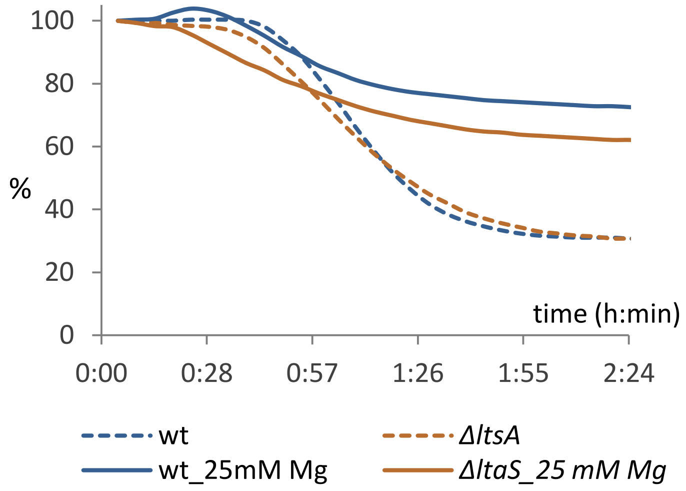

Figure S8. Wild-type and *ΔltaS* cells were grown to early exponential phase (OD_{600} 0.3) in LB medium supplemented or not with 25 mM Mg^{+2} and autolysed by addition of 75 mM sodium azide. Autolysis curves are expressed as percentage of maximum OD_{600}, at which autolysis was induced.

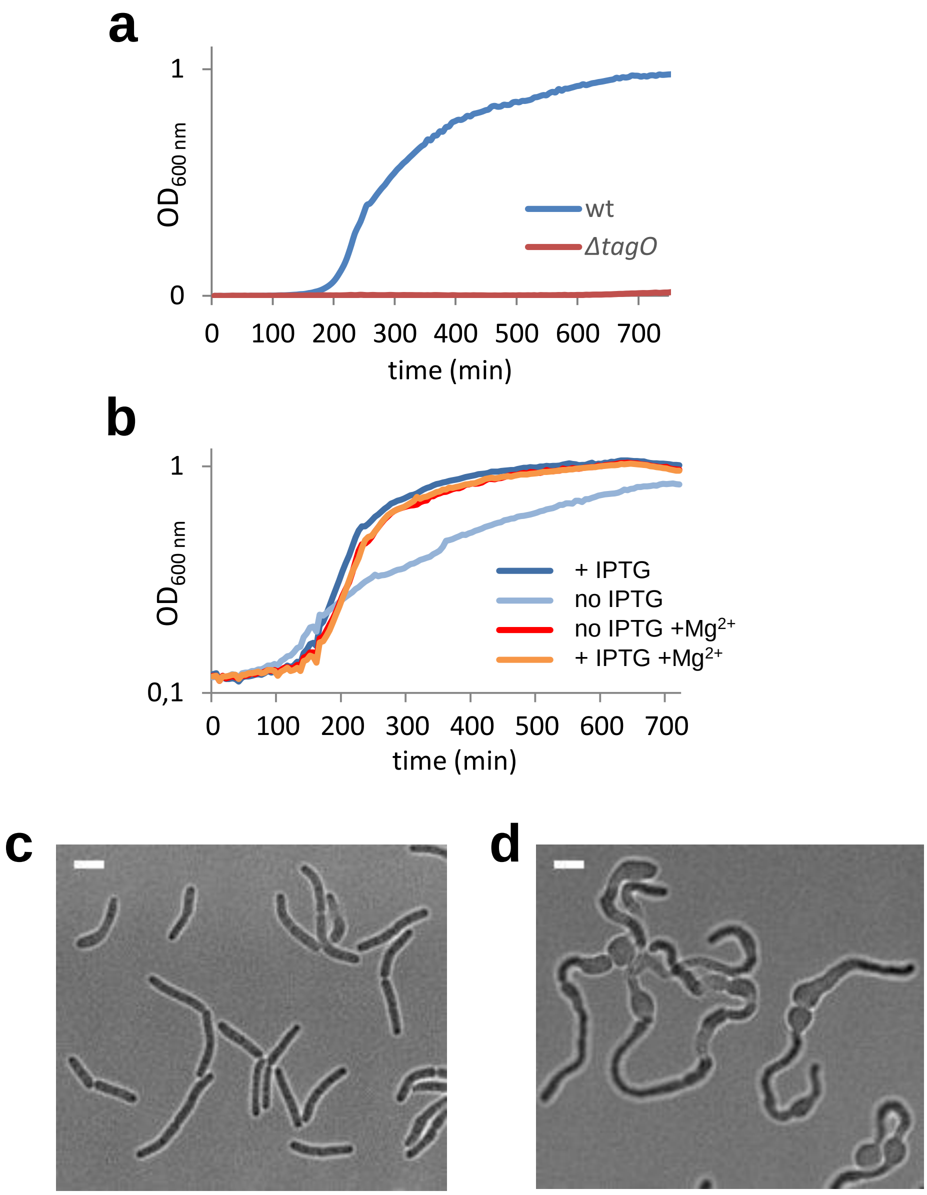

Figure S9. (**a**) Growth curves of the wild-type and the *ΔtagO* null mutant grown in unsupplemented LB medium. (**b**) Growth curves of the P_{spac}*tagO* strain grown in LB containing or not 0.5 mM IPTG and 25 mM $MgSO_4$. (**c, d**) Phase contrast images of P_{spac}*tagO* cells growing exponentially in LB in the absence of induder, with (c) or without 25 mM Mg^{+2} (d). Scale bars, 3 µm.

Movies Legends

Movie 1. TDL depletion experiment of Δ*mreB* cells. Cells growing exponentially in LB medium were stained with 1mM TDL for 20 min, rinsed and further grown in LB in the microfluidics device. Phase contrast (*Left*) and TDL fluorescence (*Right*) images were taken every 5 min. The first frame of the movie corresponds to 0 min after rinsing. Scale bars, 2 µm.

Movie 2. TDL depletion experiment of wild-type cells. Cells growing exponentially in LB medium were stained with 1mM TDL for 20 min, rinsed and further grown for 60 min in LB in the microfluidics device. Phase contrast (*Left*) and TDL fluorescence (*Right*) images were taken every 5 min. The first frame of the movie corresponds to 0 min after rinsing. Scale bars, 2 µm.

Movie 3. Growth of the Δ*mreB* strain in the microfluidics device at 37°C in LB medium supplemented with 25 mM $CaCl_2$. Phase contrast images were taken every 20 s.

References

1. De San Eustaquio-Campillo, A., Cornilleau, C., Guerin, C., Carballido-López, R., and Chastanet, A. PamR, a new MarR-like regulator affecting prophages and metabolic genes expression in *Bacillus subtilis*. *PLoS One* **12**, e0189694 (2017).
2. Formstone, A., and Errington, J. A magnesium-dependent *mreB* null mutant: implications for the role of *mreB* in *Bacillus subtilis. Mol. Microbiol.* **55**, 1646-1657 (2005).
3. D'Elia, M.A., Millar, K.E., Beveridge, T.J., and Brown, E.D. Wall teichoic acid polymers are dispensable for cell viability in *Bacillus subtilis*. *J. Bacteriol.* **188**, 8313-8316 (2006).
4. Mirouze, N., Ferret, C., Cornilleau, C., and Carballido-Lopez, R. Antibiotic sensitivity reveals that wall teichoic acids mediate DNA binding during competence in *Bacillus subtilis*. *Nat. Commun.* **9**, 5072 (2018).
